# Dynamic Light Scattering and Its Application to Control Nanoparticle Aggregation in Colloidal Systems: A Review

**DOI:** 10.3390/mi15010024

**Published:** 2023-12-22

**Authors:** Jesus Rodriguez-Loya, Maricarmen Lerma, Jorge L. Gardea-Torresdey

**Affiliations:** 1 Environmental Science and Engineering Ph. D. Program, University of Texas at El Paso, El Paso, TX 79968, USA; jarodriguez79@miners.utep.edu (J.R.-L.); mpalma@miners.utep.edu (M.L.); 2 Department of Chemistry and Biochemistry, University of Texas at El Paso, El Paso, TX 79968, USA

**Keywords:** theory DLVO, colloids, zeta potential, control agglomeration, chemically processed toner

## Abstract

Colloidal systems and their control play an essential role in daily human activities, but several drawbacks lead to an avoidance of their extensive application in some more productive areas. Some roadblocks are a lack of knowledge regarding how to influence and address colloidal forces, as well as a lack of practical devices to understand these systems. This review focuses on applying dynamic light scattering (DLS) as a powerful tool for monitoring and characterizing nanoparticle aggregation dynamics. We started by outlining the core ideas behind DLS and how it may be used to examine colloidal particle size distribution and aggregation dynamics; then, in the last section, we included the options to control aggregation in the chemically processed toner. In addition, we pinpointed knowledge gaps and difficulties that obstruct the use of DLS in real-world situations. Although widely used, DLS has limits when dealing with complicated systems, including combinations of nanoparticles, high concentrations, and non-spherical particles. We discussed these issues and offered possible solutions and the incorporation of supplementary characterization approaches. Finally, we emphasized how critical it is to close the gap between fundamental studies of nanoparticle aggregation and their translation into real-world applications, recognizing challenges in colloidal science.

## 1. Introduction

A colloidal system is a mixture of microparticles in a fluid medium that could be gas, water, or air. The first colloidal system dates back to 1774 when Juncher and Macquer mixed “extremely finely divided gold in a fluid” [1]. Graham, in 1861, marked the beginning of the systematic research on colloidal systems, introducing the concept of colloids, indicating the distinction between the solutions that passed through a membrane and those with no diffusion through a membrane. Currently, colloids are considered the ones that diffuse through the membrane for particles in the size range of 1 to 1000 nanometers. In 1827, Robert Brown, a Scottish botanist, detected the irregular motion of the colloidal-sized particles of pollen grain in water, now known as Brownian motion, later identified with thermal movement by Jean Perrin in 1910 [1]. Colloids are microscopic particles of various substances dispersed throughout a continuous medium of metals, polymers, and biological materials. Colloids contain a wide variety of particles with different sizes, shapes, and purposes, including artificial nanoparticles and biological components [2]. 

In contemporary times, colloids have extensive applications in everyday life, industry, and new product development that continue to grow at an unprecedented rate. Colloids have various applications, accounting for 70% of all industrial processes [3,4,5]. We can find colloids in food products such as dairy products, or some during food preparation [6,7,8,9]. They are also found in the manufacturing process of pigments, as a thickening agent in lubricants, lotions, toothpaste, and coatings [10], and other applications. Some pharmaceuticals are colloids [11], which allow them to be easily absorbed by the human body, making them more efficacious [12,13,14]. The development and knowledge of colloidal science faces a considerable challenge regarding controlling and influencing nanoparticles’ agglomeration forces in a suspension system. One exciting application using DLS involves sizing nanomaterials in situ for metal oxide nanoparticles (MONs) [15]. A great variety of systems have a propensity for the particles to aggregate or cluster, which may substantially impact the system’s characteristics and behavior [16,17,18]. Therefore, understanding and controlling aggregation processes in colloidal systems is essential for commercial and scientific purposes [19,20,21].

From a great variety of research to handle the particle’s aggregation forces, some have a significant [22,23,24,25,26,27,28,29,30,31,32,33,34,35,36], electrostatic interactions [24,27,29,37,38], solvent conditions [39,40], particle size and shape [41,42] critical influence: surface chemistry, and external fields such as electric or magnetic fields [43,44]. The chemical composition of the particle surface can strongly influence aggregation behavior [45]. For example, particles with hydrophobic surfaces aggregate more readily than those with hydrophilic surfaces [46]. Particles with charged surfaces can either repel or attract each other, depending on the type and magnitude of the charges. Controlling the particles’ surface charge can be an effective way to manipulate their aggregation behavior [47]. The solvent environment can have a significant impact on particle aggregation [48,49]. For example, adding salt to a colloidal solution can screen electrostatic repulsion, increasing the cluster [50,51]. The size and shape of particles can also affect their aggregation behavior [47]. For example, elongated particles may align in specific orientations, leading to ordered aggregates [52]. Applying external fields, such as electric or magnetic fields, can also influence particle aggregation [53]. These fields can induce particle alignment or motion, leading to ordered aggregates.

Various experimental and theoretical techniques exist to study and control aggregation processes in colloidal systems. For example, small-angle X-ray scattering (SAXS) [54] and DLS [55,56,57,58] are commonly used to characterize particle size and aggregation behavior. Computer simulations, such as Monte Carlo [59,60,61,62] and molecular dynamics simulations [63,64,65,66,67], can also provide insight into the fundamental mechanisms of particle aggregation. SAXS has some restrictions for use in a practical environment, such as complex data analysis requiring complicated mathematical calculations to extract information [54,68,69,70]. Computer simulation using Monte Carlo modeling involves significant computational resources, and model validation can be challenging, as it does not capture all aspects of real-world systems [56,71]. On the other hand, DLS is easier, faster, and well-suited to studying diluted systems and is, therefore, more accessible in the manufacturing industry or research labs than the other techniques [56]. Each colloidal system has specific parameters depending on its components, such as raw material properties (chemical composition), mixing speed and heat control method, temperature, particle size and shape, and the components’ heterogeneity.

Because a colloidal system involves many variables, it can be challenging to control aggregation processes. Brownian motion randomly affects colloidal particles in a fluid due to collisions with solvent molecules and other particles. This irregular kinetic movement can lead to the impact and subsequent aggregation of particles, making it not easy to control cluster sizes. Electrostatic interactions are another critical force in colloidal particles, which can lead to attractive forces between particles and promote aggregation. These interactions are difficult to control, especially in complex systems where the electrostatic forces are affected by pH, salt concentration, particle shape, particle size, mixing speed, mixing interactions, and whether it is a homogeneous or heterogeneous system (different components), among other factors. Colloidal particles also interact through Van der Waals forces. These forces are attractive at short distances and can promote aggregation, but they rapidly decrease as the particles are separated, making it hard to control aggregation. Colloidal systems can be heterogeneous, with particles of different sizes, shapes, and surface chemistry. This heterogeneity can lead to complex aggregation behavior, making it difficult to control the process.

Due to the rising usage and applications of colloids in many industries, better methods are required to manage the forces in a colloidal agglomeration system. Understanding how all the agglomeration forces interact in a colloidal system is critical. However, developing a practical method or technology to help achieve this target is far from daily life’s applicable terms. The current review aims to find a workable real-time approach to monitoring a colloidal system’s electrical conductivity or ionic strength. Besides the general background on the other control agglomeration techniques, the focus is the application of DLS to control the pace of the colloidal aggregation at a specific particle size target and narrow particle size distribution.

We developed a search strategy for this literature review to identify the relevant literature. This search strategy was tailored to three databases: Scopus, ScienceDirect, and MinerQuest, and the search terms used were the following: “controlled agglomeration” and “dynamic light scattering”. All searches spanned from the databases’ inception from 2018 to 2023, including journal articles, review papers, and research reports published in English only.

**Figure 1:**
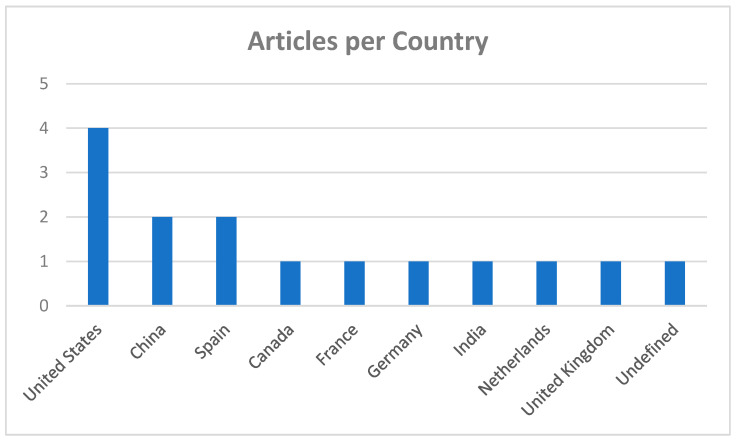
Relevant articles per country. The United States has a higher number of articles per country from the years 2018–2023 relating to control agglomeration.

**Figure 2:**
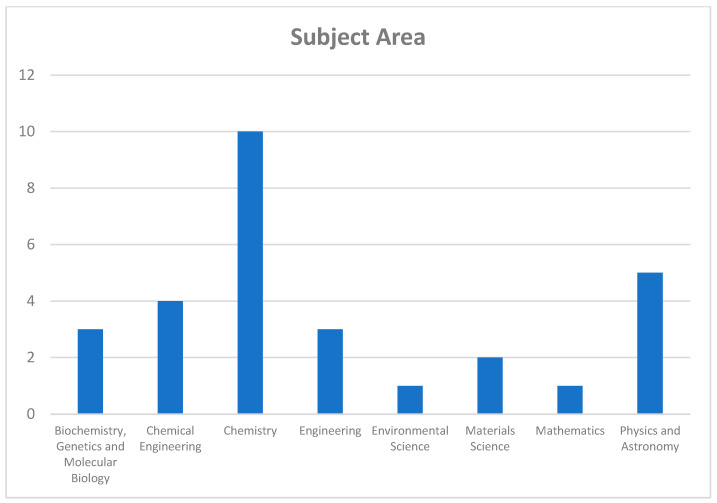
This figure depicts the breakdown of relevant articles published between 2018 and 2023 concerning controlled agglomeration, categorized by their respective subject areas. As shown, the field of Chemistry dominates the distribution with the highest number of articles published, followed by Physics and Astronomy. This suggests a significant focus on the application of controlled agglomeration within the realm of chemical research.

The selection criteria were based on the PRISMA statement [72]. The search mainly mapped the existing literature on “controlled agglomeration” and “dynamic light scattering” in chemical engineering, materials science, environmental science, and chemistry. The search then narrowed to the subject areas of colloidal science. The primary focus of this literature review was on articles after 2018, with just a few exceptions, including a deep-diving review of the 14 latest research articles. Figure 1 and Figure 2 summarize the main countries and areas related to this article.

## 2. Dynamic Light Scattering: Particle Size, Zeta Potential, and Molecular Weight

**Figure 3:**
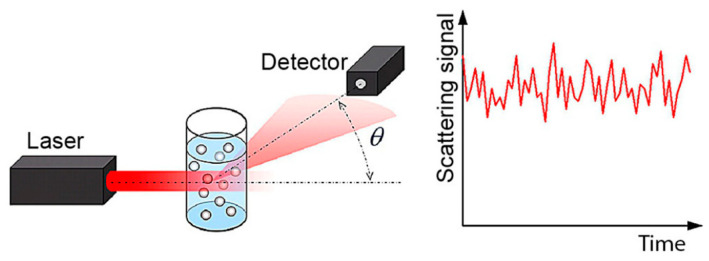
DLS principle of operation: laser light hits particles in suspension, and a high-time-resolution detector measures the intensity of the scattered light at a specific angle *θ*; scattered light intensity is due to particles’ Brownian motion fluctuating over time. Reproduced with permission from reference [73].

Scattered light from suspended particles in a colloidal suspension gives information on the sample’s physical properties (Figure 3). These suspended particles in the solution have continuous movement due to Brownian motion; if an electrical potential is applied, the particles achieve a specific velocity. Radio frequency used in a radio detection and ranging (RADAR) system was developed in World War II [11]. The same principle was applied to develop DLS, applying the Doppler effect to identify the position and velocity of particles. DLS uses the light of laser Doppler velocimetry (LDV) to measure a particle’s velocity. RADAR analyzes the Doppler shift in radio waves reflected from moving objects to determine an object’s velocity, whereas scattered laser light experiences a Doppler shift in frequency.

Due to the random mobility of the liquid-borne particles, their intensity changes over time when they scatter light. The size of the particles in a sample, ranging from a few nanometers to several microns in diameter, can be verified using DLS by examining these variations. On the other hand, zeta potential measures the particles’ stability in suspension by quantifying the particle’s electrical charge. Particles with high zeta potential reject each other and stay in suspension, while those with low zeta potential tend to agglomerate or flocculate. Combining those three parameters causes them to complement each other for better dispersion or particle-in-solution characterization.

### 2.1. Particle Size Theory

The essential operation of DLS to measure particle size is based on the Brownian motion of particles in suspension. The random speed motion of particles in a liquid depends on the particle’s size. Small particles move quickly, and large particles move slowly. DLS, also known as photon correlation spectroscopy (PCS), relates the Brownian motion to particle size by using a laser to illuminate the particles and analyze the intensity variations in the scattered light. The Stokes–Einstein equation describes the connection between a particle’s size and the speed it experiences because of Brownian motion [74].

**Figure 4:**
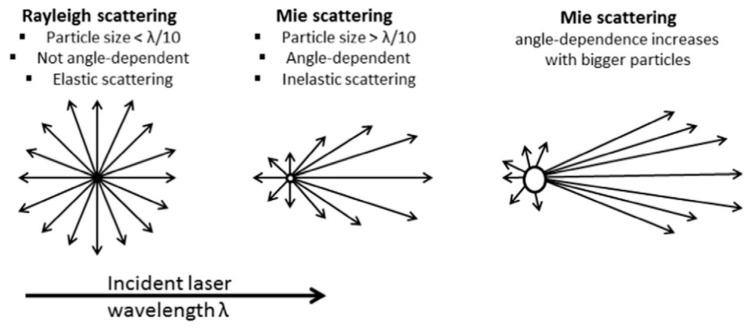
Incident light that strikes dispersed nanoparticles (NPs) is scattered to the 6th power of NP’s radii. Particle size <1/10th of the incident wavelength (λ/10), scatter light with same energy (Rayleigh scattering). Particle size >1/10th of the incident wavelength scatter light is angle dependent (Mie scattering). Reproduced with permission from reference [76].

Figure 4 depicts the scattered light beams as the laser rays hit the particles. Dispersed nanoparticles (NPs) scatter incident light according to their radii to the sixth power. Particles with a wavelength <l/10th of the incident light in size produce elastic scattering (scattered light with the same energy as the incident beam), known as Rayleigh scattering. When a particle size >l/10 creates inelastic scattering or Mie scattering (scattered light with different energy from the incident beam) [75], particles are constantly in motion, causing the destructive and constructive phase addition of the scattered light appearing to fluctuate in intensity. DLS measures the rate of intensity fluctuation and then uses this to calculate the size of the particles. DLS has installed a digital correlator that measures the degree of similarity between two signals over a specific time. The time for the correlation to decline to zero in a typical speckle pattern is 1 to 10 milliseconds. Large particles move slowly, and then the speckle intensity fluctuates gradually. While small particles move quickly, their speckle intensity fluctuates quickly. DLS uses the correlation function to calculate the size distribution. DLS software uses algorithms to obtain the decay rates for several size classes to produce a size distribution. One of the typical graphs is the distribution graph, where the *x*-axis shows the size class distribution.

The size intensity distribution generated by DLS is converted to volume distribution using Mie theory. Volume distribution can be converted to a number distribution. However, the intensity distribution is the primary measurement for obtaining the other measures, based on the intensity of scattering of a particle, which is proportional to the sixth power of its diameter, from Rayleigh’s approximation.

### 2.2. Molecular Weight Theory

Dynamic light scattering used to measure the size of particles or molecules in a solution is related to hydrodynamic radius and molecular weight. DLS requires combining other techniques to give an accurate molecular weight determination. The techniques, such as size exclusion chromatography (SEC) and multi-angle light scattering (MALS), can give a more reliable and precise molecular weight [77]. Moreover, DLS requires known molecular weight calibration standards to estimate molecular weight.

Static light scattering (SLS) and DLS can be combined in specific ways to estimate the molecular weight of macromolecules in a solution, including polymers or proteins. Multi-angle light scattering (MALS) or static light scattering paired with DLS are used to describe this combination technique [78]. The particles in a sample are lit by a light source, such as a laser, and the particles scatter the light in all directions, like dynamic light scattering, which is used to determine particle size. However, static light scattering uses the time-averaged intensity of scattered light rather than the time-dependent oscillations in the scattering intensity.

For various sample concentrations, the intensity of light scattered over time—roughly 10 to 30 s—is accumulated. “Static Light Scattering” refers to removing the signal’s intrinsic oscillations caused by this time averaging. For several sample concentrations, the natural scattered light fluctuations over a period are averaged, eliminating its natural oscillations known as SLS. The laser beam illuminates the sample, measuring the time-averaged intensity of the scattered light. From here, it determines the molecular weight and the second virial coefficient (A2). A2 describes the interaction’s strength between the particles and the solvent.

A2 > 0, the particle’s high affinity to the solvent.

A2 < 0, the particle’s low affinity to the solvent tends to aggregate.

A2 = 0, the strength of particle–solvent interactions is equal to the strength of molecule–molecule interactions.

In static light scattering, DLS applies the Rayleigh equation that describes the scattered light intensity from a particle in a solution at different concentrations, as described in Equation (1).

(1) $$\frac{K.C.}{R_{\Theta }}=(\frac{1}{M_{W}}+2A_{2}C)P(\Theta )$$

$R_{\Theta }$ = Rayleigh ratio—the ratio of scattered light to incident light of the sample

$M_{W}$ = Sample molecular weight

$A_{2}$ = Second virial coefficient

C = Concentration

P(Θ) = Scattering angle

K = Optical constant, calculated in Equation (2):

(2) $$K=\frac{{4\pi }^{2}}{\lambda_{0}^{4}N_{A}}\times {\left(n_{0}\frac{dn}{dc}\right)}^{2}$$

$N_{A}$ = Avogadro constant

$\lambda_{0}$ = Laser wavelength

$n_{0}$ = Solvent refractive index

$\frac{dn}{dc}$ = Differential refractive index as a function of the change in concentration. If not available in the literature, it can be measured using a differential refractometer.

The molecular weight is measured by comparing a well-defined pure ‘standard’ scattering intensity with a known Rayleigh ratio to the analyte.

The accepted method for determining molecular weight is to measure the analyte’s scattering intensity compared to a well-defined ‘standard’ pure liquid (e.g., toluene) with a known Rayleigh ratio.

The scattered light intensity produced by a particle is proportional to the product of the weight-average molecular weight and the concentration of the particle.

The appropriate molecular measurement weight range for one DLS is from a few hundred g/mol to 500,000 for linear polymers and over 20,000,000 for proteins and near-spherical polymers.

DLS evaluates the intensity of scattered light of different sample concentrations at a single angle; this value is contrasted with the scattering caused by a standard (i.e., toluene).

### 2.3. Zeta Potential Theory

**Figure 5:**
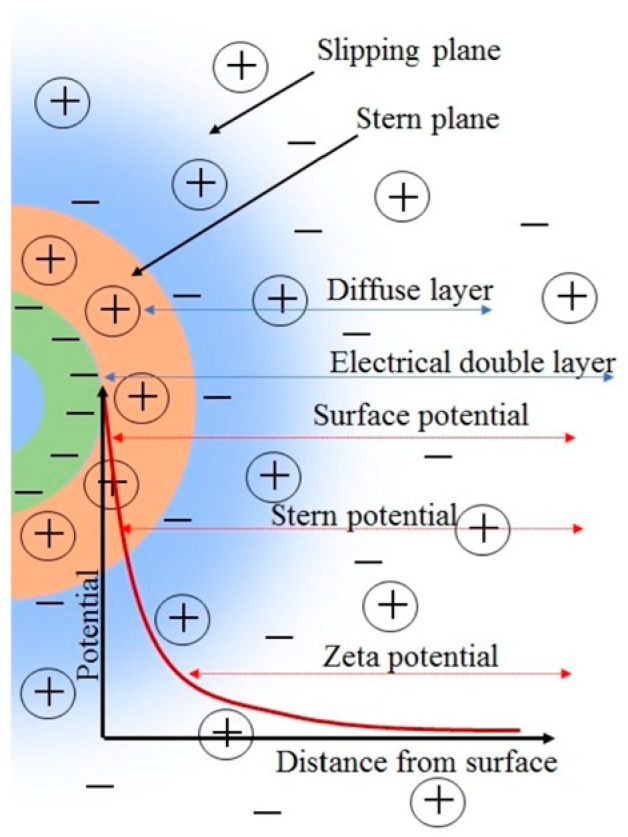
Zeta potential and the electrical double layer. Reproduced with permission from reference [76].

Zeta potential is an excellent way to evaluate the electrostatic stability of suspensions by measuring the particle’s charge at the shared or slipping plane (Figure 5).

**Figure 6:**
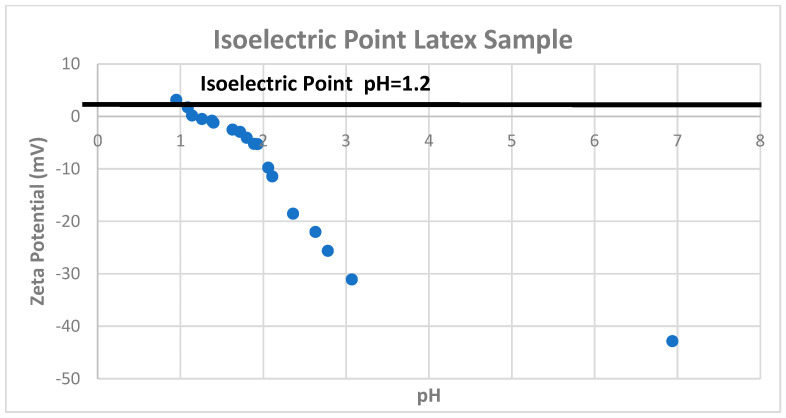
Isoelectric point latex sample.

Zeta potential is the electrical potential developed at the solid–liquid interface [40,75,76,77,79,80,81,82] and depends on several factors [83]. Some factors depend on solvent parameters, such as pH, conductivity, temperature, presence, type, and concentration of ions. These parameters control particle mobility, including agglomeration, dispersion, coalescence, coagulation, and separation [76]. pH is one of the most critical elements in a solution [84,85] where the isoelectric point (IEP) is affected depending on the concentration of H^+^ and OH^−^ ions (Figure 6). Differences in the IEP in the same material indicate stability issues where the colloidal system is less stable. The electric double layer’s (EDL) thickness depends on the ion concentration and valency; a higher concentration compresses the double layer. Increased ionic concentration boosts conductivity but can decrease particle mobility; therefore, special care should be taken when choosing suspension parameters.

DLS can deduce zeta potential in suspension particles by measuring charged particles’ electrophoretic mobility. DLS uses laser Doppler velocimetry (LDV) to measure the particle’s velocity. LDV defines the particle’s velocity with zeta potential, first evaluating the electrophoretic mobility ($\mu_{e}$) (Equation (3)):

(3) $$\mu_{e}=V/E$$

V=particle velocity (μm/s);

E=electric field strength (Volt/cm).

From the previous equation, replacing $\mu_{e}$ in Henry’s equation (Equation (4)):

$\varepsilon_{r}=relative\;permitivity/(dielectric\;constant$);

$\varepsilon_{0}=permitivity\;of\;vacuum$;

$f\left(Ka\right)=Henry^{’}s$ function;

Ꞇ = zeta potential;

*ƞ* = viscosity.

Zeta potential Ꞇ can be calculated using the following equation:

(4) $$\mu_{e}=2\varepsilon_{r}\cdot \varepsilon_{0}Ꞇf(Ka)/3\eta$$

$\varepsilon_{r}=relative\;permitivity/(dielectric\;constant)$;

$\varepsilon_{0}=permitivity\;of\;vacuum$;

$f\left(Ka\right)=Henry^{’}s\;function$;

Ꞇ=zeta potential;

η = viscosity.

If the electric double layer (EDL) is much smaller than the particle radius due to larger particles < 1 μm in high-salt-concentration aqueous solutions of (10^−2^ M), $f\left(Ka\right)$ is equal to 1.5, modifying Henry’s equation to the Helmholtz–Smoluchowski (HS) equation (Equation (5)).

(5) $$\mu_{e}=\varepsilon_{r}\cdot (\varepsilon_{0}Ꞇ)/\eta$$

When the thickness of the EDL is much larger than the particle radius due to smaller particles (≤100 nm) in low salt concentrations (10^−5^ M), $f\left(Ka\right)$ is equal to 1, and Henry’s equation is modified as per the Huckel equation (Equation (6)).

(6) $$\mu_{e}=2\varepsilon_{r}\cdot \frac{{\varepsilon_{r}\varepsilon }_{0}Ꞇ}{3\eta }$$

It is always recommended to report the zeta potential value and its current pH. It is a way of indicating the expected predominant particle charge; a low pH means the particle’s positive charge, or vice versa. Zeta potential +/− 30 mV is generally accepted as the general dividing line between stable and unstable suspensions. A value closer to zero is less stable. Higher values of +/− 30 mV indicate stable dispersions. However, Kamble et al. [76] describe good stability (Table 1) for suspensions with zeta potential between +/− 40 and +/− 60.

An important concept for evaluating the stability of dispersions is the isoelectric point, the pH point where the colloidal system is least stable. Some other significant effects under electrical fields are the electrokinetic effects. These effects depend on the particle’s induced motion: electrophoresis, electroosmosis, streaming, and sedimentation potential. Electrophoresis is the particle’s velocity while applying to an electric field. Significant factors in this measurement are the electric field’s strength or voltage gradient, the dielectric constant, the viscosity of the medium, and zeta potential.

It is important to reiterate that, for the most common Henry’s function in aqueous media with a moderate electrolyte concentration (i.e., particles > 0.2 microns and 10^−3^ molar salt), the Smoluchowski approximation is 1.5. In systems with small particles in low-dielectric-constant media, Henry’s function becomes 1.0.

## 3. DLS and Its Applications

DLS is a widely utilized technique in many scientific and industrial sectors where the distribution of particle size and behavior of particles in solutions is crucial. With the recent appearance of well-known lipid nanoparticle (LNP)-based products, such as the SARS-CoV-2 vaccines from Pfizer, Inc.-BioNTech (BNT162b2), and Moderna, Inc. (mRNA-1273), its application to nanoparticles in medicine is becoming more and more significant [11,86]. DLS is crucial for the characterization and quality assurance of vaccines and treatments based on nanoparticles. This article describes some fields where DLS has extensive applications. Having the capacity to measure particle size and distribution, besides measuring zeta potential and some other colloidal properties, such as molecular weight, makes DLS a practical device for various applications.

### 3.1. Pharmaceutical Industry and Human Health

In the pharmaceutical industry, DLS has applications for formulating the development of new medicaments, nanoparticle aggregation research, and protein characterization [13,14,86]. For instance, DLS helps evaluate protein stability during the screening and characterization of drug candidates in the development of biopharmaceuticals [14]. For evaluating blood stability [87], zeta potential is a crucial metric in describing the electrostatic interaction between red blood cells in dispersed systems; therefore, it is critical to avoid hemagglutination, which is highly important in blood transfusion [76] and blood storage [88]. In some cases, there are applications in combination with other techniques, like the use of Raman spectroscopy with DLS in a single platform for the characterization of therapeutic proteins at high concentrations [13] to monitor protein aggregation. Similar studies mention DLS using gold nanoparticles (Au NPs) for biomolecular detection, bioimaging, drug delivery, and photothermal therapy [89]. For instance, zeta potential can be a practical guide when determining the equilibrium between the drug’s positive and negative charges to ensure adequate uptake and release phenomena [76,90,91]. DLS is used to evaluate gold nanorods–protein interaction, and its characterization demonstrates that DLS is a valuable tool [87,92].

Particle size (PS) and particle size distribution (PSD) have wide applications in various fields. Particle size is a crucial characteristic influencing cellular absorption, biodistribution, and drug release profile [11]. For instance, controlling particle size in lipid nanoparticle (LNP)-based products used in the manufacturing of SARS-CoV-2 vaccines from Pfizer, Inc.-BioNTech (BNT162b2), and Moderna, Inc. (mRNA-1273) is a critical parameter [11,72]. Hassett et al., in mice experimentation [93], report that the hydrodynamic diameter of LNPs affects their biodistribution, stability, and circulation rate across the body and their cell absorption. LNPs act as delivery systems for mRNA vaccines, encapsulating and introducing the mRNA antigen into cells. Lipids, the building blocks of these LNPs, self-assemble into nanoscale particles to protect and insulate the delicate mRNA molecules. The consistency and stability of these lipid nanoparticles are crucial to the vaccinations’ effectiveness [94,95] vaccinations’ effectiveness. Particle and protein aggregation is highly studied in the pharmaceutical sector, and DLS is a practical instrument that helps to control this property.

Another instance of using DLS in the pharmaceutical industry is in manufacturing magnetic nanoparticles, which have interesting medical applications for developing sensing and diagnostic systems. Lim et al. studied the size distribution and colloidal stability of magnetic nanoparticles (MNPs) using DLS [96]. The authors mentioned that MNP with Fe^0^ and Fe_3_O_4_ can be an effective nanoagent to remove pollutants from water.

### 3.2. Material Science

The application of DLS in the characterization of colloids, nanoparticles, and polymers in material science is extensive. Development and process control in the industries of paints, pigments, food and beverages, cosmetics, ceramics, and personal care products are some fields where DLS has some advantages over destructive tests, such as microscopic imaging, electrical sensing (Coulter) counters, hydrodynamic or field flow fractionation, disc centrifuge particle sizing, size exclusion chromatography, and scattering techniques, among others [96,97]. Lim et al. [96], using DLS, studied size distribution and colloidal stability of magnetic nanoparticles (MNPs). The authors contrasted DLS with transmission electron microscopy (TEM) and dark-field microscopy [96], revealing both the advantages and disadvantages of DLS in measuring the size of MNPs. Specifically, zeta potential has applications in electrophoretic deposition (EPD) in the preparation of advanced materials [98] and the separation of minerals using water, such as flotation, where the wetting of the mineral surface is affected by the oriented water layers on the solid surface, as well as the EDL to control the flotation process with the point of zero charge (PZC) as a critical parameter [76].

### 3.3. Environmental Protection, Remediation, and Toxicology

**Figure 7:**
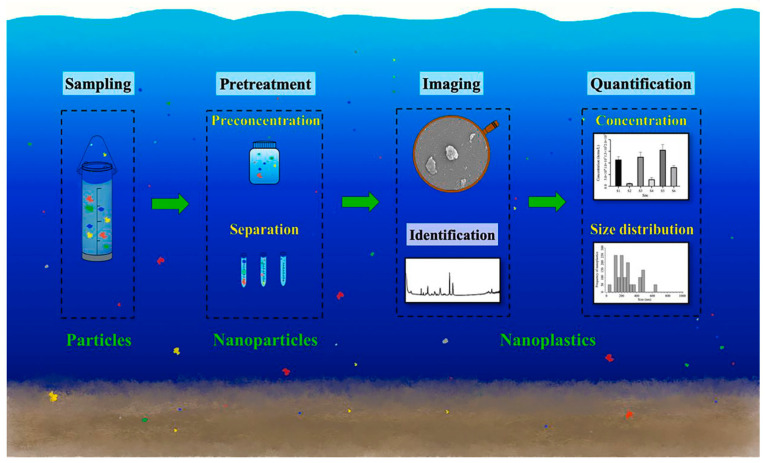
Analyzing nanoplastics in environmental samples. Reproduced with permission from reference [99].

Some applications of DLS in measuring particle size are in environmental protection. Cai H. et al. [99] applied ultrafiltration, DLS, pyrolysis, thermodesorption, and thermochemolysis coupled to chromatography/mass spectrometry (GC-MS) to separate, preconcentrate, quantify, and identify nanoplastics (Figure 7).

DLS helps characterize anthropogenic or natural organic materials, colloids, and particles on Earth’s surface, including air, water, or soil [100], and its applications in nanoecotoxicology and environmental sciences. Tareq et al. mention interesting applications where DLS measurements detected nanomaterials’ presence in Tennessee’s river waters [101]. Even though the particle’s properties, such as size, vary according to the analytical measurement method or equipment used [102,103], combining and comparing results from other methods and determining the most reliable for your process is recommended.

Mylon et al. [51] used the technique of DLS to measure the aggregation kinetics of a model virus, bacteriophage MS2, and identified the elements that make viruses unstable in aquatic environments. Mylon et al. describe the significance of biophysical interactions between viruses and their surfaces as a method to develop disinfection or viral eradication techniques. Among other exciting applications of DLS analysis is producing biofuel from algae to optimize the separation of algae mass from water; zeta potential plays an important role where charged algae cells are generated in contact with water [76]. Our current development and technological progress generate excessive nanoparticle releases into the environment, demanding the close monitoring of air and water pollution. One option is using DLS to detect nanoplastics in various products for human consumption [104].

Using DLS, Shahid et al. explained how to measure the hydrodynamic radius for extracting heavy metals from an aqueous solution. The authors extracted Co^+2^ ions from water using poly (N-isopropylacrylamide-acrylamide-methacrylic acid) p(NAM) as an adsorbent [105].

Overall, DLS can be utilized to monitor water and air quality by analyzing suspended particles and aggregates in water or air samples [106]. Moreover, DLS can be employed to study suspended particles in soils or sediments [107]. It contributes to understanding soil composition, structure, and possible environmental effects by determining particle sizes, their distribution, and their interactions.

### 3.4. Food Science

DLS has applications in characterizing food colloids, emulsions, and suspensions. Tosi et al. [108] used DLS to quantify particle/molecular sizes, particle size distribution, and relaxations in complex fluids for food applications. They exposed the accuracy of this quantification, which is critical in evaluating the toxicity and exposure level of nanoparticles in foods. Evaluating quality, process control measures, and composition is beneficial in producing milk products and their stability. The casein’s particle size in milk affects its flavor. Large particle sizes tend to float up, causing phase separation (creaming). If the particle size is too small, it flocculates. The electrostatic charge that the particles carry is essential for the stability of the milk emulsion. Monitoring the particle size is crucial to ensure milk satisfies consumer demands, legal requirements, and shelf-life regulations [109].

DLS is widely used for research in food science, and it plays an essential role in the characterization and evaluation of colloids, emulsions, and suspensions. Rao et al. [110] described DLS as a faster and easier technique with lower limit detection to measure particle size than other techniques. One of the advantages of DLS is that sample preparation is minimal and noninvasive. Also, dynamic measurements in situ show immediate results. However, a disadvantage is that concentration is a limiting factor in this technique since the sample to be analyzed must be highly diluted to avoid multiple scattering.

Palm oil in water microemulsion is used as a delivery system for hydrophobic nutrients. In an accelerated stability study on palm oil in water microemulsion for 28 days, DLS was used to determine the stability of the microemulsion’s membrane. After 28 days, DLS showed a reduction in particle size, which explains that the membrane suffered breakage, creating smaller droplets; therefore, the stability decreased [111].

DLS has been used in the characterization of nanoencapsulated food ingredients. In a study performed in encapsulated vitamins D3 and β-carotene in tripolyphosphate-chitosomes (TPP-Ch), at three different dilutions (100×, 500× and 1000×), DLS shows that, as dilution increases, aggregates decrease [108]. Esposto et al. [112] measured the encapsulation efficiency of α-carotene, β-carotene, and phenolic compounds in liposomes, chitosan (Ch), and TPP-Ch. DLS results showed that β-carotene had the highest encapsulation efficiency.

In summary, DLS is a useful analytical tool to characterize and evaluate food nanosystems. Also, it gives an insight into the product’s physical properties and behavior that ultimately will determine the food’s shelf life.

### 3.5. Toners, Inks, and Pigments

Another area where DLS plays an essential role due to controlled particle size and PSD is in the printing industry. Manufacturing toners and inks requires close particle size control to improve stability, printing quality, and shelf life. Inks and pigment dispersions tend to sediment, coalesce, and flocculate due to particle size changes, primarily due to particle agglomeration [113].

DLS is a widely used technique for characterizing toner particles in the toner industry. A blend of polymer resin, colorants, and magnetic particles make up the small powder known as toner used in photocopiers and laser printers.

DLS can assess the toner particle size distribution and track aggregation and size changes over time. DLS measurements can help improve the formulation and manufacturing processes by giving information about the stability of toner suspensions. The zeta potential of toner particles, a measurement of the electrostatic repulsion between particles in a solution, can also be found via DLS. Their zeta potential can impact particle stability, aggregation, and adhesion.

However, at the same time, there is a need to develop more environmentally friendly pigments. Pandian et al. used DLS in their research to propose nanopigment colorants. These pigments derive from pulp and paper industrial waste black liquor due to their lignin content [114].

## 4. Particle Agglomeration Mechanisms and Their Control in Colloidal Systems

It is essential to understand the main stability forces to describe the possible mechanism to control the agglomeration forces involved in a colloidal system.

Table 2 describes the significant forces involved in a colloidal system. Here, we can summarize the major interaction forces among particles in suspension.

Vincent et al. mentioned that after the World War II, Russian researchers Derjaguin, Landau, Verwey, and Overbeek in the Netherlands independently developed a theory that explains particle interactions in dispersions. This theory, now called the DLVO theory, was developed one hundred years after previous experiments [115]. The DLVO theory explains the stability of aqueous particle suspensions through the interactions of van der Waals and electric double-layer forces, explaining how they are stable at low salt levels and unstable at higher salt concentrations [57].

**Figure 8:**
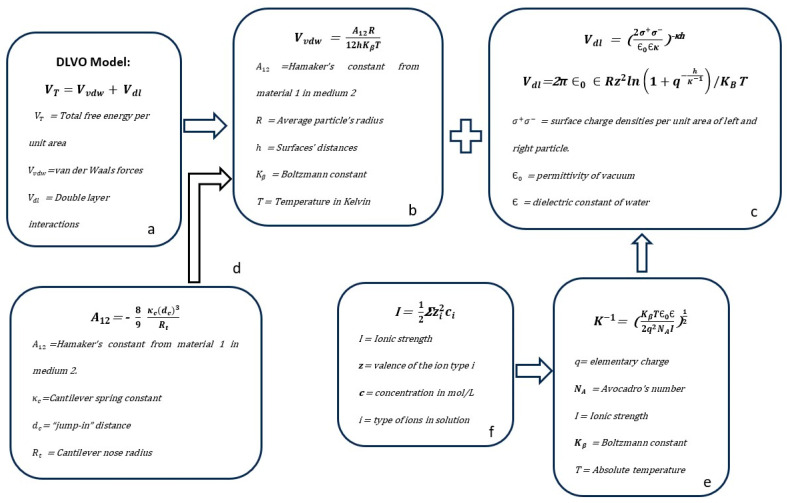
Parameters and variables in a colloidal system and their particle agglomeration performance are explained using the DLVO (Derjaguin, Landau, Vervey, and Overbeek) theory (**a**). Two main contributors to the total free energy per unit area are the additive forces of (*V_vdw_*) van der Waals and (*V_dl_*) double-layer interactions (**a**). To evaluate the *V_vdw_* forces (**b**), the Hamaker constant can be measured using the Atomic Force Microscope (AFM), as described in (**d**). The *V_dl_* forces (**c**) can be defined using any of the two equations. At the same time, the ionic strength (**f**) can be measured to replace the value in equation (**e**).

The theory behind the forces involved in the aggregation process is the DLVO model (Figure 8).

The DLVO theory is the theoretical basis of colloidal particle interactions and its agglomeration performance in colloidal suspensions developed by Derjaguin, Landau, Vervey, and Overbeek [115] Electric double layers, van der Waals, are the primary colloidal forces in a colloidal system, but other forces intervene in colloids, such as hydration and steric forces.

Two significant additive contributors, according to the DLVO theory, are:

(7) $$V_{T}=V_{vdW}+V_{dl}$$

$V_{T}$ = Total Free Energy;

$V_{vdW}$ = van der Waals Forces;

$V_{dl}$ = Double-Layer Interactions.

Attractive van der Waals forces come from the atoms’ dipoles and the molecules’ rotation or fluctuations.

(8) $$V_{vdW}=-\frac{A_{12}R}{12hK_{\beta }T}$$

*A*_12_ = Hamaker constant, material 1 in medium 2;

*R* = Particle’s average radio;

*h* = Surface distances;

$K_{\beta }=Boltzman\;constant$;

T=Temperature in Kelvin.

**Figure 9:**
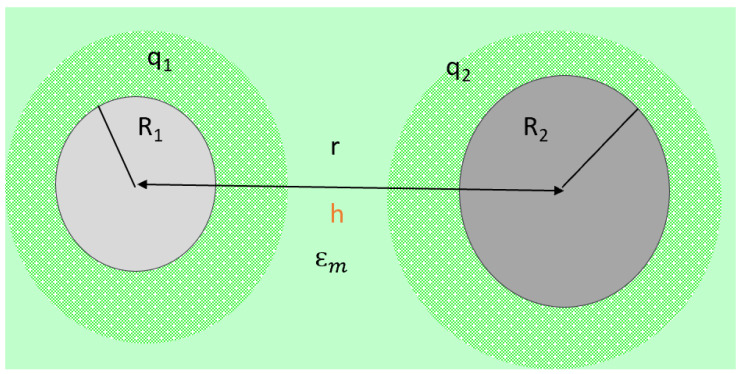
Major variables in a colloidal system: qi = particle charge, Ri = particle radius, r = surface particle distance, h = particle center distance, Єm = dielectric permittivity from the medium.

Figure 9 depicts and locates the significant variables described in the next paragraph involved in the DLVO model.

Repulsive electrostatic interactions come from the double layers of each particle. We can use Equation (9) or Equation (10).

(9) $$V_{dl}=\frac{2\sigma^{+}\sigma^{-}}{\in_{0}\in K}Exp(-Kh)$$

(10) $$V_{dl}=2\pi \in_{0}\cdot \in Rz^{2}ln\left(1+q^{-\frac{h}{K^{-1}}}\right)/K_{B}T$$

$\sigma^{+}\sigma^{-}$ = Surface charge density per unit area;

$\in_{0}$ = Vacuum permittivity;

∈ = Water dielectric constant;

$K^{-1}$ = Inverse Debye length.

(11) $$K^{-1}={\left(\frac{K_{B}\in_{0}\in }{2q^{2}N_{A}I}\right)}^{1/2}=\frac{0.3\;nm}{\sqrt{I}}$$

*q* = Elemental charge;

$N_{A}$ = Avogadro number;

I = Ionic strength;

$K_{B}$ = Boltzmann constant;

T = Absolute temperature.

Ionic strength:

(12) $$I=\frac{1}{2}\sum z_{i}^{2}c_{i}$$

$z_{i}$ = valence of the ion type *i*;

$c_{i}$= concentration in mol/L;

*i* = type of ions in solution.

However, besides the DLVO forces, there are non-DLVO forces in a colloidal system. These could be hydration and hydrophobic forces related to water layer interactions and entropic factors [47]. Entropic changes can also result in osmotic forces, as seen when polymer-coated NPs interact via interpenetrating chains. Inter-particle repulsion is demonstrated by NP surface coatings with surfactants, polymers, and polyelectrolytes through steric, electronic, and electrostatic phenomena. An increase in temperature causes the Brownian motion of an NP suspension to increase and the water shear to decrease, which encourages aggregation. Moreover, NP concentration affects the stability of emulsions and is, therefore, one factor to consider in controlling the agglomeration rate; surface changes, including steric, hydrophobic, magnetic, and hydration factors, impact NP aggregation. Aggregation is influenced by potential surface manipulation; levels less than 20 mV may result in aggregation [47]. As can be seen, the combination of several concepts, such as point of zero charge (PZC), particle surface charge, particle surface coating, NP concentration in the system, zeta potential (ZP), critical coagulation concentration (CCC), and particle crystalline structure, can affect the aggregation rate in a colloidal system.

## 5. Particle Agglomeration Control in Chemically Processed Toner

**Figure 10:**
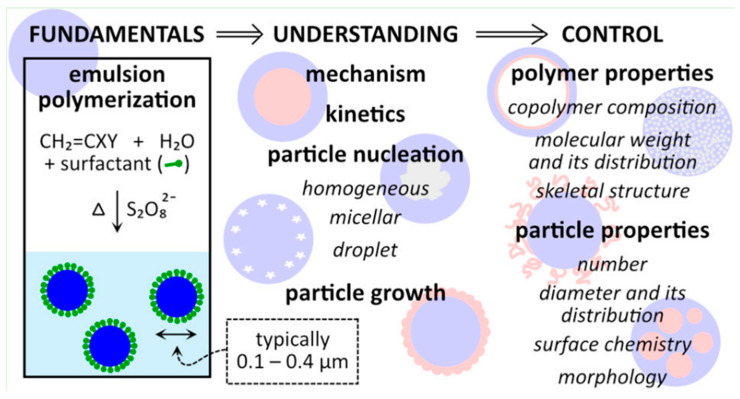
Emulsion aggregation. Nucleation types. Depicts the nucleation mechanism types in emulsion polymerization, mainly represented as free-radical polymerization [121]. Open access.

Ataeefard et al. [116,117] mentions that emulsion aggregation offers the best results for controlling agglomeration out of the four CPT toners (suspension polymerization, dispersion polymerization, emulsion aggregation, and chemical milling). Moreover, this paper states how increasing the agitation speed decreases particle size and narrows the particle size distribution. The reasoning behind this conclusion is that higher turbulence induces shear rates. The author recommends increasing the mixture speed during the acid addition, dispersing the acid component as quickly and uniformly as possible. Andami et al. [118] propose the use of in situ polymerization methods [119] (suspension, emulsion, and mini emulsion) to control the microstructure of toner particles (particle size, particle size distribution, and sphereness) with the energy level at a reasonable level in the presence of a redox precursor. The authors described the superiority of mini-emulsion compared to the other two methods, emphasizing the advantages of lower polymerization temperature and higher conversion of mini-emulsion compared to suspension and emulsion polymerization techniques. One interesting fact mentioned in this article is that using sodium formaldehyde sulphoxylate (SFS) as a reducing agent (redox initiator) in the emulsion polymerization method allowed the reaction temperature to be lowered down to 40 °C compared to suspension polymerization with a temperature of 70 °C. A solvent-free redox polymerization reaction provides a mild reaction condition responsible for high conversion [120]. The mini-emulsion copolymerization was executed with similar conditions to emulsion polymerization but at 55 °C using a cetylalcohol (CA) co-stabilizer. These three in situ polymerization types have different nucleation mechanisms, microstructures, and color characteristics. Out of the three mechanisms (micellar, droplet, and homogeneous nucleation), droplet nucleation has the highest efficiency for manufacturing toner with a spherical shape, appropriate size, and narrow size distribution (Figure 10).

During this research, we identified some exciting topics using a redox initiator in the emulsion polymerization and the mini-emulsion polymerization process to fabricate chemically processed toner [5,122]. It opens the possibility of lowering the temperature from 70 °C used in suspension polymerization to 40 °C and 55 °C for emulsion and mini emulsion, along with a 28% and 22% increase in conversion [123].

Looking into options for redox initiators in emulsion and mini emulsion polymerizations, with possible applications in the manufacturing of chemically processed toner, we found the following:

- Potassium persulfate and sodium persulfate. Potassium persulfate is the oxidation agent, while sodium bisulfite is the reducing agent. The reaction between these two compounds generates free radicals that initiate [121,124].
- Ammonium persulfate and sodium metabisulfite. Ammonium persulfate is the oxidizing agent, while sodium metabisulfite is the reducing agent. The reaction between these two compounds generates free radicals that initiate polymerization.
- Cerium(IV) ammonium nitrate can be used in mini-emulsion polymerization. Cerium(IV) ammonium nitrate is the oxidizing agent and initiates polymerization at low temperatures [118].
- Hydrogen peroxide and ferrous sulfate can be used in mini-emulsion polymerization. Hydrogen peroxide acts as the reducing agent [118].

In practice, in the stage of acid addition, there are three combinations we need to research: acid addition rate, high shear rate speed, and flow rate. These conditions must be investigated to find the optimal sequence to increase yield and reduce particle size distribution. Moreover, identifying the right reducing agent could increase the final output.

## 6. Conclusions and Future Remarks

From this deep dive into the literature on colloidal science and particle agglomeration control using DLS, it is evident that great discoveries and new knowledge have been achieved by researchers on this subject in recent years. Importantly, colloidal science influences and constantly generates new knowledge from many fields [125], such as medicine, the food industry, material science, and environmental protection, among others. Furthermore, at the same time, DLS plays an essential role in better understanding practical ways to control dispersion and suspension in our daily life tasks. Using DLS in the industry and health sector allows more sectors to control colloidal suspensions easily. However, finding the proper practical application of colloidal science in these processes in our daily lives has some challenges. The kinetics of aggregation in colloidal systems is complex, making it quite difficult to control and monitor the aggregation process, primarily considering a broad spectrum of variables involved: particle concentration and its composition, the polydispersity of the colloidal system, solvent properties, temperature, pH, and ionic strength, among others. DLS has limitations; for instance, sample preparation interferes with the measurement, and thus, one must depend on extensive adjustments to reach optimal experimental conditions. In addition, samples with high viscosity or turbidity affect the sensitivity of DLS. Achieving the optimal experimental conditions and adequately understanding the kinetics of the system under investigation are essential for improving the control of particle agglomeration in a colloidal system. Going forward, colloidal scientists must advocate their efforts to apply all the progress and improvements in this field more practically.

Nevertheless, at the same time, research societies should vigorously publicize and share innovations from colloidal knowledge that can improve the quality of life in industry and daily human activities. DLS has more potential for practical applications than most colloidal specialists know outside of their expertise. Also, there is an urgency to optimize and dedicate more research to utilize DLS methods and techniques to apply them to polydisperse solutions with high viscosity and a greater variety of temperatures. Moreover, there is a possibility of improving DLS principles and applying them in higher concentrated dispersions for reactor batches to allow for in-line measurements including other variables such as temperature, viscosity, and dispersion charge for more accurate measurements.

Overall, this review helps to close that gap and makes more people interested in extending their analytical skills using DLS and expanding their colloidal knowledge.

## Figures and Tables

**Table 1:** Suspension stability vs. zeta potential. Reproduced with permission from reference [76].

Assessment of Stability	Zeta Potential (mV)
Flocculation of coagulation	0 to +/−5
Incipient instability	+/−10 to +/−30
Moderate stability	+/−30 to +/−40
Good stability	+/−40 to +/−60
Excellent stability	Greater than +/−60

**Table 2:** Forces influencing the stability of NPs in liquid medium. Reproduced with permission from reference [90].

Force	Influence
van der Waals	Short-range electromagnetic force between NPs, attractive.
Electrical Double Layer	Electrical interaction between NPs due to the overlap of electric double layer, typically repulsive.
Hydration Force	Interaction between water molecules on hydrophilic NPs, repulsive nature.
Hydrophobic Force	Attractive interaction between hydrophobic NPs in water.
Steric, Electronic, and Electrostatic Forces	Surface coatings: inorganic, surfactants, polymers, and polyelectrolyte on NP surfaces. Polymers can form bridges leading to osmotic forces for interpenetrate chains. Surface coatings can have attractive or repulsive effects.

## Data Availability

No new data were created or analyzed in this study. Data sharing is not applicable to this article.
